# IFN‑γ Production in Memory CD4^**+**^ T Cells in Response to MSP1_19_ Antigen and Its Correlation with Anemia and Thrombocytopenia in Pediatric Vivax Malaria

**DOI:** 10.1021/acsomega.5c01165

**Published:** 2025-06-10

**Authors:** Ana C. Shuan Laco, Yury O. Chaves, Anne C. G. de Almeida, Elizangela S. Farias, Victor I. Mwangi, Marcia V. G. Vallejos, Gerhard Wunderlich, Paulo A. Nogueira, Gisely C. de Melo

**Affiliations:** † Programa de Pós-Graduação em Ciências aplicadas à Hematologia (PPGH-UEA), Universidade do Estado do Amazonas, Manaus, Amazonas 69050-001, Brazil; ‡ Programa de Pós-Graduação em Medicina Tropical (PPGMT-UEA), Universidade do Estado do Amazonas, Manaus, Amazonas 69040-000, Brazil; § Fundação Oswaldo Cruz (FIOCRUZ), Instituto Leônidas & Maria Deane, (ILMD/Fiocruz-Amazônia), Manaus, Amazonas 69057-070, Brazil; ∥ Fundação de Medicina Tropical Doutor Heitor Vieira Dourado, Manaus, Amazonas 69050-001, Brazil; ⊥ Instituto de Ciências Biomédicas da Universidade de São Paulo, São Paulo 05508-000, Brazil

## Abstract

Plasmodium vivax (P. vivax) can lead to severe hematological complications, including anemia and thrombocytopenia, which may require hospitalization. This prospective observational study investigated the adaptive immune response in pediatric P. vivax malaria, focusing on sustained IFN-γ production in CD4^+^ and CD8^+^ memory T cells as markers of immune protection. Conducted at the Dr. Heitor Vieira Dourado Tropical Medicine Foundation (FMT-HVD) in the Western Brazilian Amazon, peripheral blood mononuclear cells (PBMCs) were collected from 25 children aged 1–16 years at diagnosis (D0, acute phase) and 90 days post-treatment (D90, convalescent phase). PBMCs were stimulated *ex vivo* with the conserved P. vivax antigen, the 19 kDa fragment of the C-terminal region of merozoite surface protein-1 (*Pv*MSP1_19_), and intracellular production of IFN-γ was quantified in memory T cells (CD3^+^CD4^+^CD45RO^+^ and CD3^+^CD8^+^CD45RO^+^ T cells) by flow cytometry. Individuals whose cells specifically produced IFN-γ against *Pv*MSP1_19_ on both D0 and D90 were classified as responders, while those who responded in only one phase or in neither were classified as nonresponders. Responders, whose CD3^+^CD4^+^CD45RO^+^ T cells exhibited sustained IFN-γ response against *Pv*MSP1_19_ demonstrated lower parasite density as well as improved normalization of hemoglobin levels and recovery of platelet counts. These results confirm the role of this response as acquisition of immunity. *Pv*MSP1_19_ antigen emerges as a promising marker of sustained immunity, though larger studies are required to evaluate its long-term impact on recovery and outcomes. These findings underscore the critical role of IFN-γ-producing memory T cells in controlling parasitemia and mitigating anemia and thrombocytopenia in pediatric P. vivax malaria.

## Graphical abstract

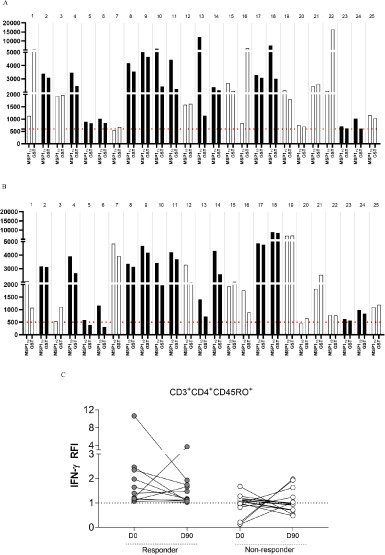

## Introduction

Malaria is a complex infectious disease that remains a great challenge to global public health. Approximately 263 million new cases are detected annually in various regions globally, primarily in tropical and subtropical countries.^1^ In Brazil, about 99.9% of malaria cases occur in the Amazon region, primarily caused by P. vivax.^2^

Children under five years of age and pregnant women represent the most vulnerable groups, contributing significantly to the morbidity and mortality rates associated with the disease.^1^ *Vivax* malaria in children can lead to complications often associated with severe anemia and thrombocytopenia, conditions that may require hospitalization.^3,4^ The risk factors for these complications include age under five, high parasitemia, comorbidities, shock, and respiratory distress. An additional risk factor is the sex of the child, in which males have underlying conditions like glucose-6-phosphate dehydrogenase (G6PD) deficiency. G6PD deficiency is linked to the X chromosome, making it a direct functional impact in males.^5^ Severe anemia (hemoglobin ≤5 g/dL) and thrombocytopenia (platelets <60,000/mm^3^) warrant attention due to the risks of renal insufficiency and severe bleeding.^5,6^

In malaria, anemia is multifactorial and involves several different mechanisms. Generally, anemia arises from the destruction of both infected and healthy red blood cells, a process exacerbated by an increased spleen and immune system activity to contain the infection. Additionally, the production of new red blood cells is impaired by an inflammatory response and hindered regeneration triggered by the infection, especially in cases of recurrent infection. In endemic regions, where the risk of malaria recurrence is high, this added factor intensifies disease processes, potentially leading to greater morbidity. Early treatment is therefore essential to prevent severe complications and reduce childhood morbidity in these areas.^6–10^

Although parasitemia is typically low and the parasite is less aggressive than P. falciparum, destruction of uninfected red blood cells (RBCs) per infected cell in P. vivax infections is greater than in Plasmodium falciparum (P. falciparum) infections.^11^ Additionally, the accompanying exacerbated inflammatory response in P. vivax infection can worsen the clinical condition, particularly in children.^10,11^ The balance between pro-inflammatory and immunomodulatory cytokines is essential in determining the clinical outcome of the infection, as an excessive or uncontrolled response can aggravate the condition.^12–14^ This exacerbation promotes similar mechanisms of parasitized RBC rupture and phagocytosis of RBCs due to membrane alterations. Furthermore, immunological deficits can impair the production of new cells.^9,10^

Progression to severe malaria cases can occur when the innate immune response is insufficient to eliminate the parasite, requiring the adaptive immune system to act as a defense and to modulate inflammation to prevent excessive host injury.^3,13,15–17^ An intense inflammatory response, marked by interferon-gamma (IFN-γ), inhibits red blood cell production and promotes precursor apoptosis, thereby worsening anemia.^11,18,19^ Conversely, the adaptive immune response in controlling parasitemia can lead to excessive red blood cell removal, further destroying uninfected erythrocytes.^20^

Cytokine production plays a crucial role in controlling parasitemia and is modulated by parasite load.^3^ High parasitemia has been shown to trigger stronger immune responses that can alternate between pro-inflammatory and protective effects, whereas low parasitemia tends to induce moderate immune activation.^3^ Memory T cells are essential for sustained protection, driven in part by the continued presence and activity of IFN-γ-producing T cells. CD45RO^+^ T cells that secrete IFN-γ are considered classical markers of functional memory.^21^ The study by Wipasa et al. (2011) validated the use of *ex vivo* PBMC assays coupled with flow cytometry to quantify CD4^+^ CD45RO^+^ IFN-γ^+^ T cells. Their results demonstrated that IFN-γ responses were more durable in individuals previously infected with P. vivax compared to those with prior P. falciparum infection.^22^ A seminal and methodologically robust study using three distinct assays to assess responses to peptides from the circumsporozoite protein, focusing on IFN-γ production by CD4^+^ T cells and antibody levels, revealed substantial variability across methods and a lack of correlation with antibody responses, highlighting the need for studies targeting conserved T cell epitopes.^23^ Although the C-terminal region of MSP1 (MSP1_19_) is structurally constrained by multiple disulfide bonds, which may hinder antigen processing and limit T cell activation in some models, studies have shown that CD4^+^ T cell responses can still be elicited, particularly when the antigen is presented in unfolded or reduced forms.^24,25^ Despite these structural limitations, MSP1_19_ remains a biologically relevant target for assessing antigen-specific cellular immunity.

Although traditionally associated with parasite control, T cell-mediated immune responses may also contribute to hematological alterations during malaria. In a murine model, Safeukui et al. (2015) demonstrated that CD8^+^ T cell-dependent clearance of *Plasmodium* parasites in the spleen was accompanied by the removal of uninfected erythrocytes, resulting in significant anemia despite low peripheral parasitemia.^20^ While this model does not directly assess canonical markers of functional immunity, such as cytokine production, it provides mechanistic evidence that effective antiparasitic immunity can lead to collateral hematological effects. In light of this, evaluating the IFN-γ-producing capacity of CD4^+^ CD45RO^+^ memory T cells during acute vivax malaria may help elucidate potential associations between Th1 responses and hemoglobin decline in naturally infected children. The objective of this study was to evaluate the role of IFN-γ-producing memory T cells in response to the conserved *Pv*MSP1_19_ antigen in controlling parasitemia and recovering hematological parameters in children with P. vivax malaria. Understanding the mechanisms of sustained immunity in malaria, reflected by the ability of memory T cells to reactivate and promptly respond to new infection episodes, may provide valuable insights into their crucial role in malaria protection, particularly in parasitic control and hematological recovery.

## Results

Twenty-five (75.8%) of the initial 33 participants returned on D90. Participants had a median age of 10 years and were mostly male (76.0%). The median symptom duration was 5 days, with interquartile ranges (IQR) of 4 days (IQR25) and 7 days (IQR75). Of the enrolled participants, 56.0% reported their first P. vivax infection, while 44.0% reported previous episodes of malaria. The median parasitemia was 3217 parasites/mm^3^.

### Characterization of Anti-*Pv*MSP1_19_ Responders and Nonresponders

The anti-*Pv*MSP1_19_ cellular response was defined based on intracellular IFN-γ production in CD3^+^CD4^+^CD45RO^+^ T cells after *ex vivo* stimulation with the *Pv*MSP1_19_-GST protein and glutathione *S*-transferase (GST), measured by the mean fluorescence intensity (MFI) of the PECy7 fluorochrome coupled to anti-IFN-γ. The MFI values were determined specifically within the CD3^+^ CD4^+^ CD45RO^+^ IFN-γ^+^ gated population, allowing for a more accurate assessment of the intensity of IFN-γ production among the responding memory T cells. Individuals were considered to have an anti-*Pv*MSP1_19_ cellular response when the IFN-γ production by CD3^+^CD4^+^CD45RO^+^ cells in response to *Pv*MSP1_19_ had a higher MFI than that against GST (black bars, Figure 1A). Those with MFI under GST stimulation higher than that of *Pv*MSP1_19_ were classified as nonspecific (Figure 1A). The same response was reassessed during follow-up of the same individuals on samples collected on D90 and considered as self-control (Figure 1B).

**Figure 1:**
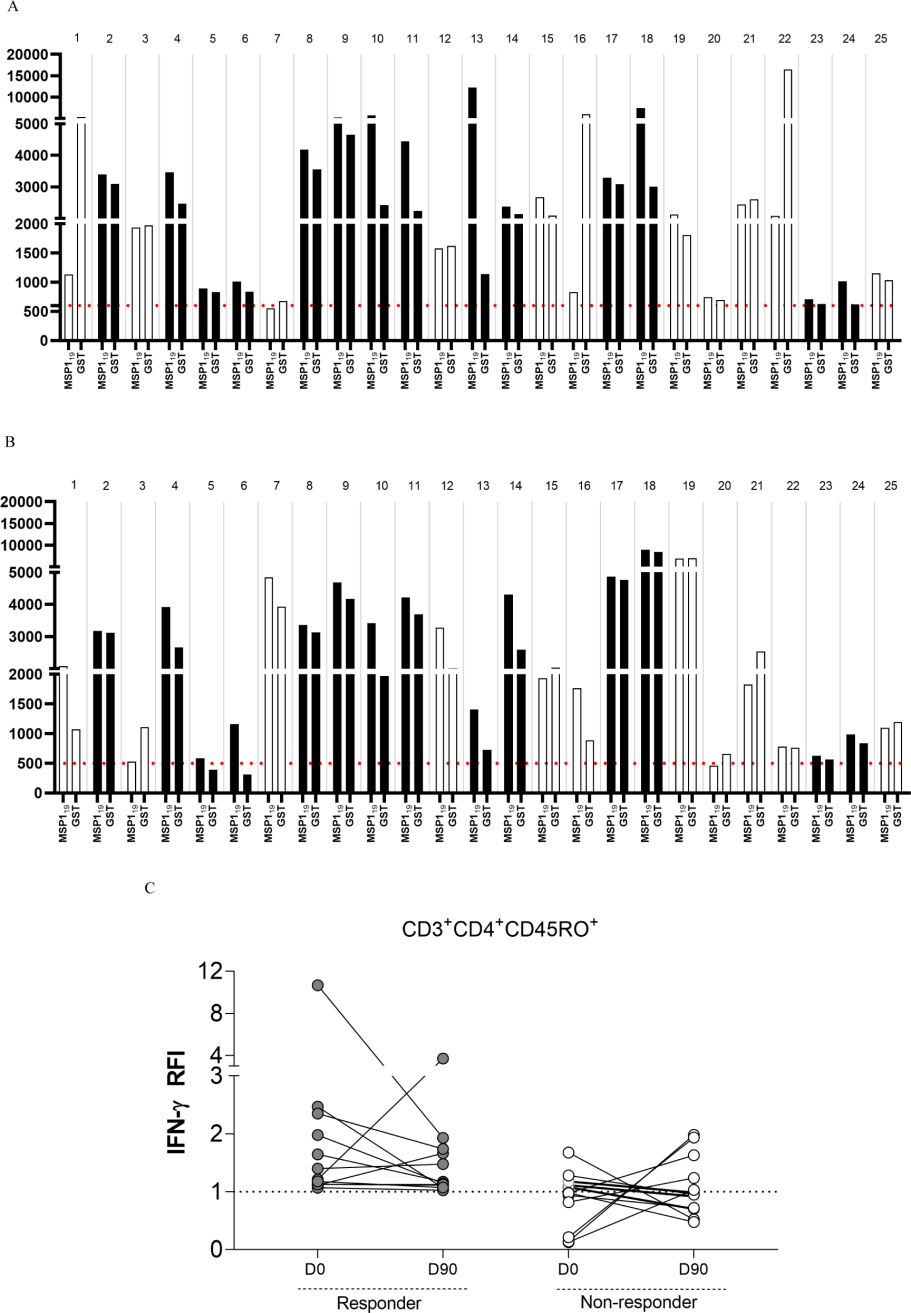
Characterization of anti-*Pv*MSP1_19_ responders and nonresponders. *Ex vivo* culture of PBMCs from 25 malaria-diagnosed patients was performed to evaluate responses to *Pv*MSP1_19_ and GST stimulation. In both (A) and (B), black bars represent only the patients with IFN-γ-producing anti-*Pv*MSP1_19_ CD4^+^ T cells in both the acute and postconvalescent phases, respectively. Intracellular IFN-γ production by CD4^+^ T cells were measured using flow cytometry, based on the median fluorescence intensity (MFI). In each pair of bars, the left bar represents the response to *Pv*MSP1_19_ stimulation, while the right bar represents the response to GST. The dashed line indicates the cutoff for baseline IFN-γ production derived from manipulation of unstimulated samples (negative control). The anti-*Pv*MSP1_19_ cellular response was defined by intracellular IFN-γ production in CD3^+^ CD4^+^ CD45RO^+^ T cells following *ex vivo* stimulation with the *Pv*MSP1_19_-GST fusion protein and glutathione *S*-transferase (GST). This was quantified by the MFI of the PECy7 fluorochrome conjugated to anti-IFN-γ. Participants were considered responders when their CD3^+^ CD4^+^ CD45RO^+^ T cells produced more IFN-γ (higher MFI) in response to *Pv*MSP1_19_-GST than to GST alone. When MFI was higher against GST than *Pv*MSP1_19_, the response was considered lacked IFN-γ-producing anti-*Pv*MSP1_19_ CD4^+^ T cells. The black bars indicate anti-*Pv*MSP1_19_ responders—i.e., individuals whose CD4^+^ T cells responded specifically to *Pv*MSP1_19_, with higher MFI for intracellular IFN-γ compared to GST, both during the acute phase and in convalescence. In contrast, the white bars represent nonresponders—i.e., participants who either lacked IFN-γ-producing anti-*Pv*MSP1_19_ CD4^+^ T cells during the acute phase, developed these memory cells only during convalescence, or failed to respond in both phases. Panel (C) illustrates an anti-*Pv*MSP1_19_ responder, based on the relative fluorescence index (RFI), where the MFI of IFN-γ in CD3^+^ CD4^+^ CD45RO^+^ IFN-γ^+^ T cells in response to *Pv*MSP1_19_-GST is greater than that in response to GST alone (i.e., RFI > 1). In gray, the plot shows that responders had RFI > 1 at both D0 and D90, while nonresponders had RFI < 1 in one or both phases (acute or convalescent).

Participants were classified as responders when *Pv*MSP1_19_-specific CD4^+^CD45RO^+^IFNγ^+^ T cells were identified on both D0 and D90 (Figure 1A,B). Of the 25 patients, 14 (56.0%) participants exhibited a specific anti-*Pv*MSP1_19_ response at both time points and thus considered responders (Figure 1A,B). The median percentage of CD4^+^ CD45RO^+^ IFN-γ^+^ cells in response to MSP1_19_ at D0 was 6.7% (IQR: 5.6%) and 5.8% (IQR: 5.8%). Of the 11 nonresponders, 2 participants remained nonresponders at D0 and D90 (Figure 1B). Cells from participants 15, 19, 20, and 25 responded only on D0 and did not sustain the response on D90. Participants 1, 7, 12, 16, and 22 showed a nonspecific response to GST on D0, but after this malaria episode, they developed anti-*Pv*MSP1_19_ immune memory by D90, responding to the *Pv*MSP1_19_ protein. These participants were classified as nonresponders due to the inconsistency in their anti-*Pv*MSP1_19_ cellular response. The relative fluorescence index (RFI) of anti-*Pv*MSP1_19_ IFN-γ and anti-GST IFN-γ by CD3^+^CD4^+^CD45RO^+^IFN-γ^+^ T cells between responders and nonresponders at D0 and D90 is shown in Figure 1C.

### Comparison of Parasite Density between *Pv*MSP1_19_ Responders and Nonresponders

Parasite density during the acute phase was compared between *Pv*MSP1_19_ responders and nonresponders (Figure 2). Individuals with anti-*Pv*MSP1_19_ CD3^+^CD4^+^CD45RO^+^IFN-γ^+^ T cells (responders) had significantly lower parasite densities than nonresponders (median 2837 [IQR 1348–5842] parasites/μL vs 8195 [IQR 4767–27,985] parasites/μL; *p* = 0.008) (Figure 2A). These findings suggest a direct association between the presence of anti-*Pv*MSP1_19_ CD3^+^CD4^+^CD45RO^+^IFN-γ^+^ T cells and their ability to control parasite density.

**Figure 2:**
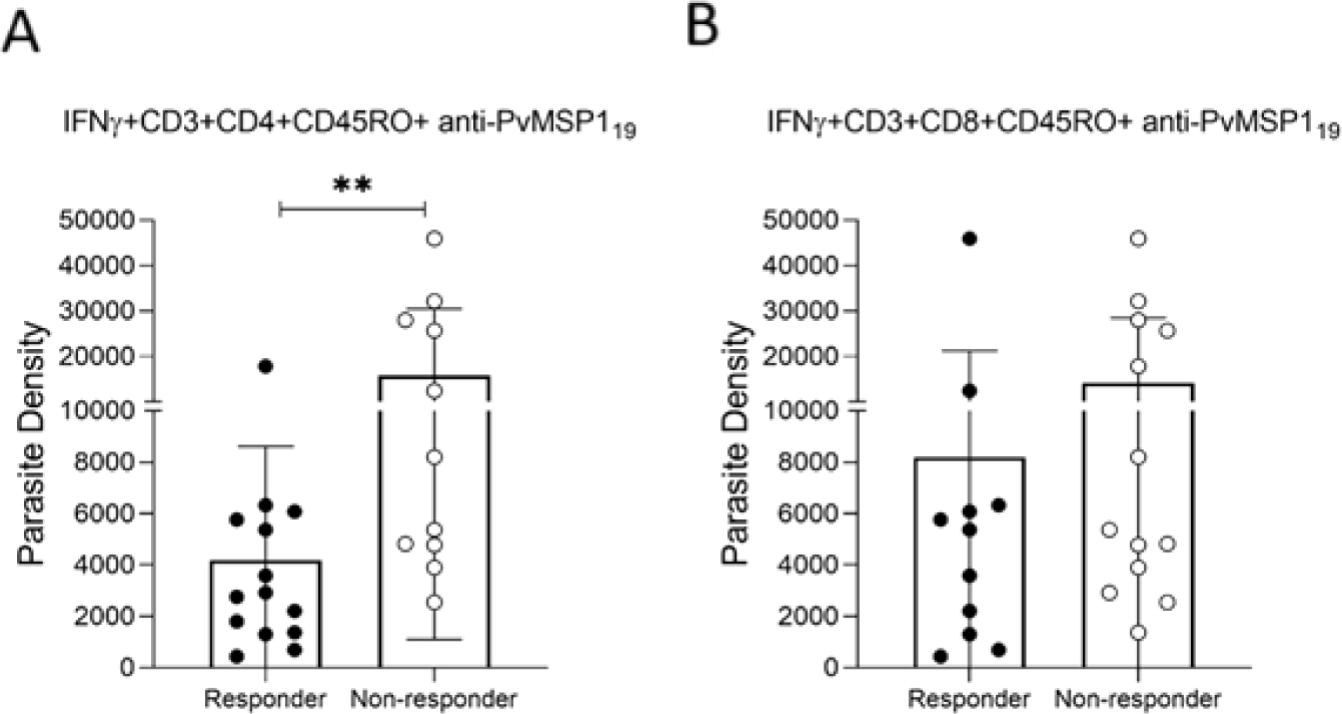
Comparison of parasitic density between responders and nonresponders. The parasitic density comparison was analyzed in parasites/μL. (A) The nonresponder group shows a significantly higher parasitic density compared to the responder group, as indicated by the asterisk (*p* < 0.05), based on a statistical comparison using the Mann–Whitney test. (B) Parasite density between responder and nonresponder groups in relation to cytotoxic CD8^+^ T cells (CD3^+^CD8^+^CD45RO^+^IFN-γ^+^ anti-*Pv*MSP1_19_) on D0 and D90, showing that the expression of these cells was not associated with a reduction in parasitemia.

In contrast, an evaluation of responders or nonresponders with cytotoxic CD8^+^ T cells (CD3^+^CD8^+^CD45RO^+^IFN-γ^+^ anti-*Pv*MSP1_19_) at D0 and D90 showed no reduction in parasitemia related to CD8^+^ T cells (Figure 2B).

### Hematological Comparison between *Pv*MSP1_19_ Responders and Nonresponders

Samples collected at D90, the postconvalescent period, allowed for comparison of hematological changes during acute malaria (Table 1). Significant recovery in red cell counts and platelets was observed at D90 in both groups (Table 1 and Figure S1). Based on World Health Organization (WHO) criteria, anemia was identified in 60.0% of participants, and 92% showed thrombocytopenia (platelets <150,000/μL) in the acute phase. No differences in hematimetric indices were observed between the acute and convalescent phases in either group.

**Table 1:** Comparison of Hematological Parameters between Responder and Nonresponder Groups in the Acute Phase (D0) and Postconvalescent Phase (D90)^a^

	Responder			Nonresponder		
Parameters (references)	D0	D90	*p* Value	D0	D90	*p* Value
Erythrocyte, 10^6^/μL^c^ ^c^ (4.2–5.5 millions/mm^3^)	4.1 (3.6–4.4)	4.7 (4.4–4.7)	0.005^b^	4.0 (3.8–4.6)	4.8 (4.6–5.3)	0.008^b^
Hematocrit, %^c^ ^c^ (36–47%)	35.1 (30.3–36.0)	38.7 (38.0–42.0)	0.002^b^	35.5 (30.4–37.2)	39.5 (38.6–43.0)	0.003^b^
Hemoglobin, g/dL^c^ ^c^ (12.5–15.5 g/dL)	10.8 (1.8)	12.3 (0.6)	0.007^b^	11.4 (1.6)	13.0 (1.1)	0.008^b^
MCV, fL^d^ ^d^ (80–100 fL)	84.1 (83.3–85.4)	84.5 (81.2–86.3)	0.940	84.0 (79.6–87.3)	83.8 (80.8–84.6)	0.860
MCH, pg^c^ ^c^ (27.0–32.0 pg)	26.3 (1.8)	26.3 (1.7)	0.980	25.1 (1.7)	26.8 (2.1)	0.053
MCHC, g/dL^c^ ^c^ (32.0–36.0 g/dL)	31.2 (2.0)	31.6 (1.6)	0.630	29.9 (1.2)	32.7 (1.6)	<0.001^b^
RDW, %^d^ ^d^ (10–15%)	13.8 (13.1–14.5)	12.7 (12.0–13.0)	0.010^b^	13.8 (12.9–15.3)	12.9 (12.0–13.2)	0.010^b^
Leukocytes, 10^3^/mm^3^ ^c^ ^c^ ^c^ (4000–10,000/mm^3^)	4842.2 (1425.6)	7469.2 (1702.1)	<0.001^b^	5050.0 (1514.5)	7345.3 (1627.1)	0.002^b^
Lymphocytes, 10^3^/mm^3^ ^c^ ^c^ (1097–2980/mm^3^)	1600.0 (1300.0–1888.0)	2400.0 (2000.0–3100.0)	0.024^b^	1100.0 (700.0–1800.0)	2800.0 (1912.0–3100.0)	0.001^b^
Monocytes, 10^3^/mm^3^ ^c^ ^c^ (220–650/mm^3^)	530.8 (342.5)	600.0 (204.1)	0.540	458.3 (271.2)	490.0 (174.0)	0.740
Neutrophils, 10^3^/mm^3^ ^c^ ^c^ (1526–5020/mm^3^)	2393.3 (1067.6)	4069.2 (1133.9)	<0.001^b^	3836.2 (1632.0)	4308.3 (1243.3)	0.430
Platelets, 10^3^/μL^d^ ^d^ (150,000–400,000/mm^3^)	95,000.0 (46,000.0–156,000.0)	282,000.0 (259,000.0–326,000.0)	<0.001^b^	83,000.0 (52,500.0–105,000.0)	255,000.0 (250,000.0–289,100.0)	<0.001^b^
MPV (fL)^d^ (7.4–10.4 fL)	10.3 (10.1–11.0)	9.1 (9.0–9.3)	0.005^b^	10.1 (9.4–10.9)	8.9 (8.1–9.5)	0.004^b^

a Abbreviations: SD = standard deviation, IQR = interquartile range, RBC = red blood cell, MCV = mean corpuscular volume, MCH= mean corpuscular hemoglobin, MCHC = mean corpuscular hemoglobin concentration, RDW = red cell distribution width, MPV = mean platelet volume.

b *p* < 0.05 values were considered statistically significant.

c Variables described with mean and standard deviation (SD).

d Variables described with median and interquartile range (IQR). Student’s *t* test was used for parametric variables, while the Mann–Whitney test was applied for nonparametric variables.

Leukocyte counts did not differ between responders and nonresponders at D0 (Table 1). On D90, levels of leukocytes and lymphocytes decreased, which did not indicate recovery, as their values remained within normal reference ranges, as during the acute phase (Table 1 and Figure S1). Monocyte levels remained consistent in both phases and were also within the normal limits. Neutrophil levels were significant between D0 and D90 in the responder group but remained within normal reference limits.

#### Normalization of Hemoglobin Levels and Platelet Recovery between Responders and Nonresponders in the Postconvalescent Phase

Changes in the acute phase were exclusively in red blood cell and platelet counts; however, although anemia and thrombocytopenia were prevalent, no participant experienced severe symptoms. As shown in Table 1, hemoglobin levels and platelet counts were restored with D90 in both groups. We further assessed the hemoglobin level and platelet count recovery in individuals within each group, as well as the individual recovery between groups.

Figure 3 illustrates the recovery of hemoglobin levels and platelet counts from D0 to D90 in both responders and nonresponders. For hemoglobin, 11 out of 14 responders presented with values below the normal range (indicative of anemia) during the acute phase, whereas only 3 out of 11 nonresponders exhibited reductions consistent with anemia at D0. By D90, hemoglobin levels had improved in all participants, with most individuals returning to values within or near the normal range (Figure 3A). Although both groups showed proportional gains over time, their recovery trajectories differed: anemia was more frequent and more severe among responders at baseline, reflecting a more substantial initial decline in hemoglobin levels.

**Figure 3:**
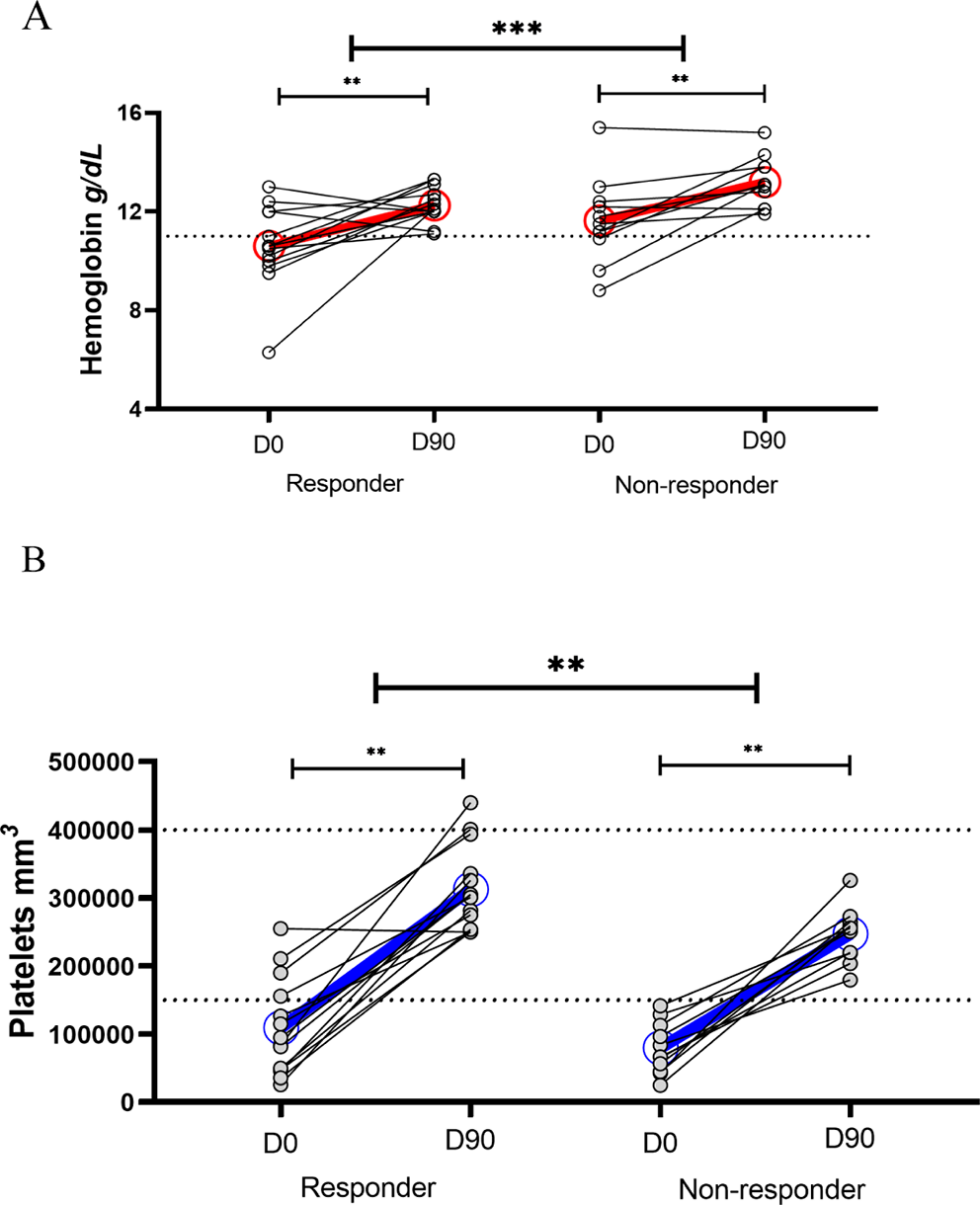
Hemoglobin and platelet recovery from acute infection (D0) to convalescence (D90) in IFN-γ responder and nonresponder groups. (A) Hemoglobin levels (g/dL) at D0 and D90 in responders and nonresponders. At D0, 11 out of 14 responders exhibited anemia (below the normal range), whereas only 3 out of 11 nonresponders were anemic. Although hemoglobin levels returned to within or near the normal range in all participants by D90, responders started from lower values and experienced a more pronounced initial drop. Consequently, their final hemoglobin levels remained relatively lower than those of nonresponders. The GEE model confirmed a significant association between IFN-γ-producing CD4^+^ T cells and greater hemoglobin loss during the acute phase (coefficient = – 0.156; *p* = 0.001). The red line connects the mean hemoglobin values at D0 and D90 to illustrate the average trend in recovery, which was steeper among responders due to lower baseline values. (B) Platelet count (×10^3^/mm^3^) at D0 and D90 in responders and nonresponders. At D0, all nonresponders exhibited thrombocytopenia, with platelet counts below the normal range, while 11 out of 14 responders were also thrombocytopenic. By D90, platelet counts had improved in all participants, returning to values within or near the normal range. Although both groups showed similar recovery slopes, the key distinction lies in the fact that thrombocytopenia was universal among nonresponders, whereas a subset of responders maintained normal platelet levels at baseline. The blue line connects the mean platelet values at D0 and D90 to illustrate the average reduction, which was more pronounced in nonresponders compared to responders, and to provide a clearer representation of platelet recovery dynamics. The GEE model revealed a significant coefficient (0.142; *p* < 0.001), indicating that responders experienced a less pronounced reduction in platelet counts. This suggests that IFN-γ production may play a role in mitigating platelet loss during acute infection. Each point represents an individual patient’s paired hemoglobin or platelet levels for D0 and D90. A nonparametric paired Wilcoxon test determined that there was a statistically significant increase after 90 days (*p* < 0.01) in both groups, for both hematological parameters. The GEE model used for intergroup comparison indicated significant differences between responders and nonresponders for both hemoglobin and platelet counts.

To assess longitudinal changes in hemoglobin levels within and between groups, we used the generalized estimating equation (GEE) model, which accounts for repeated measures in the same individuals (i.e., D0 and D90). While the average slope of hemoglobin recovery over time appeared similar between responders and nonresponders, the model revealed a significantly more pronounced reduction in hemoglobin levels among responders at baseline (coefficient = −0.156, *p* = 0.001; 95% CI: −0.241 to −0.0714). This finding is consistent with the visual data in Figure 3A, which presents a summary plot with mean trajectories of hemoglobin levels at D0 and D90 for both responders and nonresponders. The red lines connecting the group means illustrate and facilitate understanding of the overall trend, highlighting that the majority of responders experienced a sharper decline in hemoglobin, with 11 out of 14 becoming anemic at D0, compared to only 3 out of 11 nonresponders. Thus, the distinction between the groups lies not in the rate of hemoglobin recovery itself but in the greater initial loss and higher prevalence of anemia among responders. These results suggest that IFN-γ production is associated with a more pronounced decrease in hemoglobin during acute infection. Although hemoglobin levels returned to normal in both groups by D90, the deeper initial loss among responders led to relatively lower, but still normal, final hemoglobin levels when compared to nonresponders. As a consequence, while both groups reached recovery, nonresponders tended to have higher final hemoglobin values due to having started from a less critical baseline.

Figure 3B illustrates platelet recovery dynamics, showing that all nonresponders experienced thrombocytopenia during the acute phase (D0), with platelet counts falling below the normal range. Among responders, 11 out of 14 were also thrombocytopenic at D0; however, a subset maintained platelet levels within the normal range at baseline. By D90, platelet counts had improved in both groups, returning to within or near the normal range. Although the overall recovery trend was similar, the key distinction lies in the universality of thrombocytopenia among nonresponders, as opposed to the partial preservation of platelet levels observed in responders. To statistically evaluate differences in platelet recovery trajectories, we applied a generalized estimating equations (GEE) model, which is appropriate for repeated longitudinal data (D0 and D90). The model yielded a significant coefficient of 0.142 (*p* < 0.001) indicating that the reduction in platelet counts was less pronounced among responders. Figure 3B displays platelet counts using an analogous summary plot format, where blue lines connect the mean values at both time points, further underscoring the more pronounced reduction observed in nonresponders relative to responders. This positive association suggests that IFN-γ production may contribute to more efficient control of thrombocytopenia—that is, the IFN-γ response observed in responders might be associated with reduced peripheral platelet consumption. Taken together, the restoration dynamics of both hemoglobin and platelets reveal distinct hematological recovery patterns between responders and nonresponders.

## Discussion

Malaria caused by Plasmodium vivax in children can lead to severe anemia and thrombocytopenia—conditions that, when associated with renal failure, often require hospitalization.^5,6^ The development of adaptive immunity through repeated exposure plays a crucial role in controlling parasitemia and modulating the inflammatory response that contributes to these outcomes. Nevertheless, while such exposures are key to immune maturation, they may also incur hematological costs, particularly in children, including anemia resulting from cumulative red blood cell loss and inflammation-induced disruption of erythropoiesis.^20,26–30^ In this study, we used the *Pv*MSP1_19_ antigen to assess adaptive immunity by measuring the level of IFN-γ production in CD4^+^ and CD8^+^ memory T cells during acute infection and convalescence. Individuals who sustained IFN-γ-producing CD4^+^ memory T cell responses in both phases exhibited lower parasitemia, suggesting a protective role of prolonged cellular immunity.^31–34^ However, T cell-mediated responses, although traditionally associated with parasite control, may also contribute to hematological alterations during infection. These findings highlight the potential of sustained IFN-γ responses in memory T cells as a marker of effective adaptive immunity, although further studies involving severe P. vivax cases are needed to validate this association.

Similar findings have been reported in P. falciparum malaria, where IFN-γ production by PBMCs in response to infected red blood cells was associated with a reduced incidence of high-density parasitemia and clinical episodes, suggesting a role in parasite control.^35^ Unlike that study, which used whole-parasite stimulation to broadly capture immune responses to erythrocytic-stage antigens, we employed the recombinant *Pv*MSP1_19_ antigen to assess antigen-specific memory responses. Although concerns have been raised about the unfolded or reduced conformations of *Pv*MSP1_19_ potentially impairing antigen processing and T cell activation,^24^ our data show that the *Pv*MSP1_19_-GST fusion protein effectively induced IFN-γ-producing CD4^+^ CD45RO^+^ memory T cells in individuals naturally exposed to P. vivax. Memory T cells are crucial for sustained protection driven by the continuous presence and activity of IFN-γ-producing cells. In this context, the ability of *Pv*MSP1_19_ to stimulate such responses highlights its potential role in promoting prolonged and targeted immune activity, particularly relevant for modulating adaptive responses during persistent infections. While we did not assess cytokine levels over time to confirm sustained production, the presence of these responsive memory T cells suggests preserved functional capacity upon antigenic restimulation. These cells are widely recognized as markers of functional memory^21^ and Wipasa et al. (2011) reported that IFN-γ responses to P. vivax are more persistent than those to P. falciparum when assessed *ex vivo* by PBMC stimulation and flow cytometry.^22^ In our cohort, children whose memory CD4^+^ T cells responded to *Pv*MSP1_19_ with IFN-γ production exhibited lower parasitemia, indicating a possible association between antigen-specific cellular memory and reduced parasite burden. Although this does not establish a direct protective effect, these findings support the use of *Pv*MSP1_19_ as a valuable immunological marker and provide insight into mechanisms potentially involved in modulating parasitemia in P. vivax infections.

While IFN-γ-producing memory T cells appear to play a beneficial role in parasite control, accumulating evidence suggests that their activity may also contribute to hematological alterations during malaria episodes.^36^ To investigate this interplay, our study compared hematological profiles and cellular immune responses during the acute and convalescent phases in children naturally infected with P. vivax. Evidence from the Brazilian Amazon shows that even in moderate-transmission areas, P. vivax can cause significant reductions in hemoglobin across a broad age range, particularly affecting young children and women of reproductive age.^37^ Although children are generally more susceptible to malaria-related complications, we observed no severe hematological abnormalities aside from thrombocytopenia and mild to moderate reductions in hemoglobin during the acute phase. These findings may reflect the epidemiological context of our study, conducted in a low-endemicity setting where P. vivax predominates—distinct from tropical regions with higher transmission intensity, such as Papua New Guinea, Indonesian Papua, and parts of Southeast Asia, where frequent relapses and early-life exposure contribute to a greater burden of severe P. vivax-associated anemia.^9,38^ All participants showed clinical improvement and normalization of hematimetric indices during convalescence, with no evidence of recurrent hemolysis or dyserythropoiesis.^39^ These outcomes align with previous findings in similar endemic settings and may also reflect the effectiveness of the antimalarial treatment administered.^5,6,9,23,32,33^

Revisiting the potential trade-off between immune-mediated parasite control and hematological outcomes, our findings suggest that IFN-γ-mediated adaptive immune responses may impair erythropoiesis during acute malaria.^36^ It is well established that P. vivax infection disrupts the normal erythropoietic cycle through systemic inflammation, leading to ineffective red blood cell production.^9,10^ In our cohort, hemoglobin reduction was more pronounced among children who mounted robust IFN-γ responses to *Pv*MSP1_19_, even in the context of low parasitemia, suggesting that the immune response itself may contribute to anemia. This paradox echoes previous models in which antiparasitic immunity, while effective in limiting infection, also results in collateral tissue effects. For instance, Safeukui et al. (2015) demonstrated in a murine model that CD8^+^ T cell-mediated parasite clearance in the spleen led to bystander removal of uninfected erythrocytes, causing anemia despite controlled parasitemia.^20^ In addition, IFN-γ has been shown to suppress hematopoietic stem cell self-renewal and impair the function of downstream erythroid progenitors, thereby contributing to ineffective erythropoiesis during inflammatory responses.^40^ Although we did not directly assess apoptosis or bone marrow suppression, the association between robust IFN-γ responses and hemoglobin decline in our cohort suggests that IFN-γ-producing memory T cells may contribute to transient anemia through mechanisms beyond parasite clearance. Further studies incorporating longitudinal cytokine profiling and bone marrow analysis are warranted to elucidate the immunohematological pathways involved and their clinical relevance.

Thrombocytopenia in P. vivax malaria is less well understood than in P. falciparum malaria, despite being equally common. It is thought to result from a combination of mechanisms, including platelet destruction and consumption due to inflammation.^41–45^ In a study conducted in the same endemic area as adults, thrombocytopenia was associated with immune modulation, specifically the reduction of Th1 cytokines (IL-2 and IL-12) along with a constant inflammatory process marked by increased IL-1β, a pro-inflammatory cytokine.^46^ Here, we observed distinct behaviors between responders and nonresponders, with a greater reduction in platelet count during the acute phase and less marked recovery in the nonresponder group, unlike hemoglobin levels. According to a systematic review and meta-analysis, children with severe P. vivax malaria are at higher risk of severe thrombocytopenia compared to adults, suggesting that immunity levels impact the severity and recovery of platelet counts.^47^ Our findings indicate, first, that the mechanisms driving platelet and hemoglobin reduction differ. Second, the immunity level in responders, characterized by sustained IFN-γ responses in memory T cells, is associated with less platelet loss in the acute phase and greater recovery in the postconvalescent period compared to nonresponders. Taken together, our data reveal that sustained IFN-γ-mediated adaptive immune responses, driven by memory T cells, support platelet recovery, suggesting distinct mechanisms for thrombocytopenia and anemia and reinforcing the importance of adaptive immunity in controlling P. vivax malaria.

This study has several limitations. First, the small sample size may restrict the generalizability of the findings, especially given the immunological and parasitological variability across different populations. Second, external factors such as diet, nutritional status, micronutrient intake, coinfections, and preexisting comorbidities were not controlled and may have influenced both hematological recovery and immune parameters. Third, the immunological analysis focused exclusively on the response to the *Pv*MSP1_19_ antigen, which likely does not encompass the full complexity of the adaptive immune response to P. vivax, including humoral components, additional cytokines, and other cell populations. Moreover, follow-up was limited to 90 days, which restricts our ability to draw conclusions regarding the long-term trajectory of immune memory and hematological recovery. Fourth, a key limitation of this study is the absence of technical replicates performed on different days, which would have allowed for a more precise definition of intra-assay variability and the establishment of a minimal threshold for biologically meaningful differences in IFN-γ production. Although responses were measured in duplicates and a stringent cutoff was calculated using GST reactivity as the control antigen, we acknowledge that some borderline responses, where the IFN-γ levels to *Pv*MSP1_19_ do not diverge substantially from those to GST, may fall within the margin of technical variation. The retrospective design of the study and limited sample volumes prevented the repeated testing of individual samples. Future prospective studies incorporating longitudinal sampling and technical replicates will be essential to refine the classification of responders and confirm the reproducibility of subtle immune response patterns. Finally, the study was conducted in a low-transmission region of the Amazon, where P. vivax predominates. While this setting limits extrapolation to high-transmission areas, it enhances the relevance of our findings to similar endemic contexts.

## Conclusions

In conclusion, our study demonstrates that the adaptive immune response mediated by sustained IFN-γ production from memory T cells is associated with the control of parasitic density and platelet recovery in children with P. vivax malaria. These findings suggest that adaptive immunity modulation may play a crucial role in reducing hematological complications, such as anemia and thrombocytopenia, which are common in this vulnerable population. The choice of the *Pv*MSP1_19_ antigen enabled a specific and robust assessment of the immune memory response, highlighting its potential as a protective marker for persistent infections. Further studies are needed to understand the complexity of this relationship and its impact on the clinical management of P. vivax malaria.

## Methods

### Study Participants, Data Collection, and Blood Samples

This was a prospective observational study conducted at the Dr. Heitor Vieira Dourado Tropical Medicine Foundation (FMT-HVD), a reference center located in Manaus city, Amazonas state, in the western Brazilian Amazon. From July 2021 to September 2022, 120 children aged 1–16 years were diagnosed with symptomatic P. vivax malaria and treated at the FMT-HVD outpatient clinic.

Only patients who agreed to participate in the study with a confirmed diagnosis of P. vivax monoinfection were considered, while those who did not return after 90 days were excluded from the study. Of the 120 children, 33 met the inclusion criteria of the study, but 25 remained until 90 days after the malaria episode (Figure 4). The day 90 (D90) sample served as a negative control for each participant.

**Figure 4:**
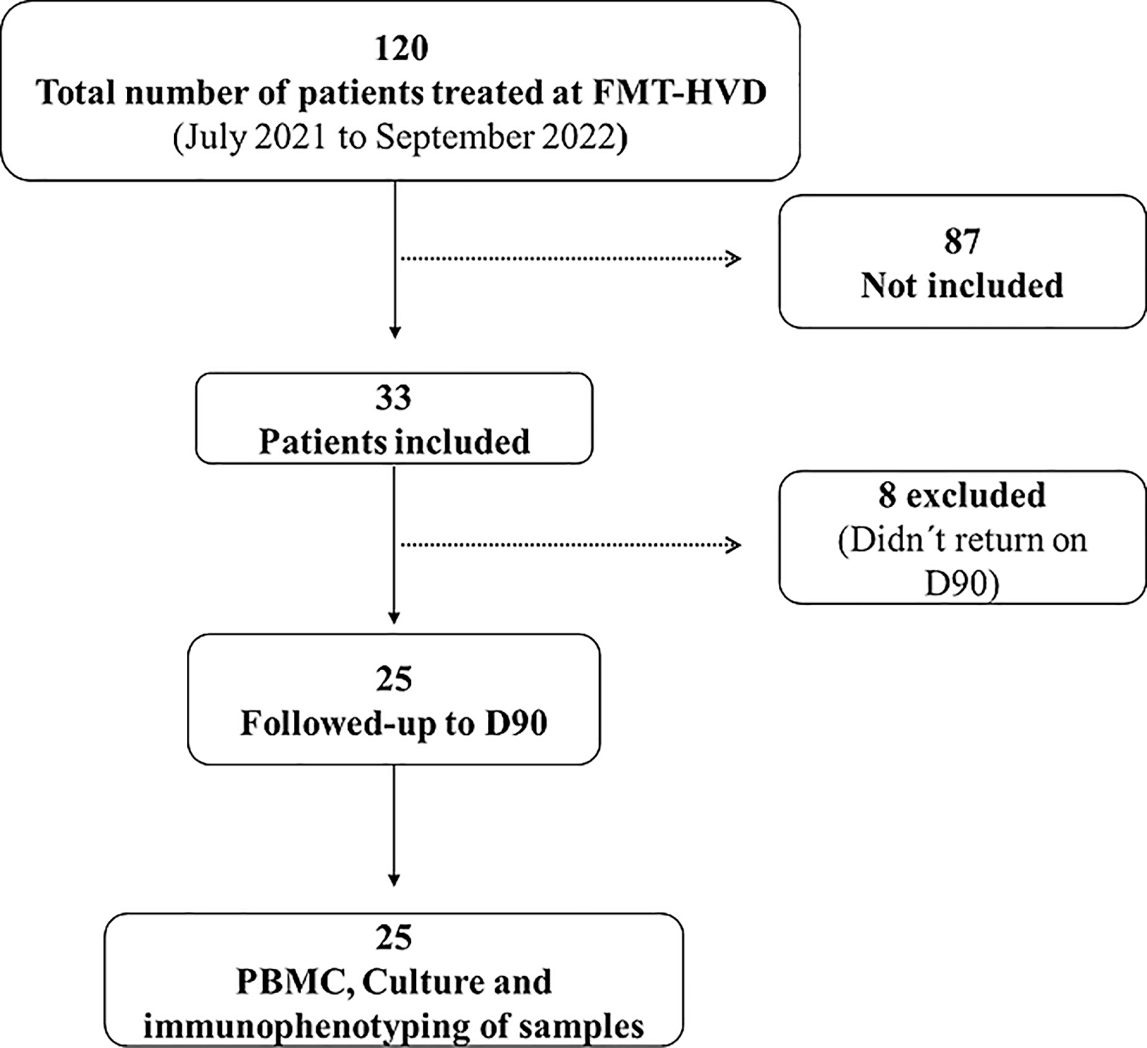
Flowchart of the selection and inclusion of participants.

The diagnosis of malaria at FMT-HVD was performed using a thick blood smear test for each patient. Slides were stained following the Walker method, and parasitemia quantification was conducted through the microscopic counting of parasites relative to 200 leukocytes observed on the slide. Parasite density was calculated by determining the ratio of counted parasites to leukocytes and multiplying it by the total leukocyte count obtained from the hemogram. Parasites were counted in 200 leukocytes, and parasitemia was quantified per mm^3^.^48^ Briefly, parasitemia was estimated by light microscopy using Giemsa-stained thick blood smears based on the number of parasites counted per 200 white blood cells (WBCs). Parasite density (parasites per microliter of blood) was calculated using the formula: (number of parasites counted ÷ 200 WBCs) × total WBC count/mm^3^. When individual WBC counts were not available, a standard reference value of 8000 WBCs/mm^3^ was used.

Blood samples were collected on the day of diagnosis (D0: acute phase) and again several weeks postinfection (D90: convalescent phase). At both time points, 9 mL of blood were collected in a heparinized tube (BD Vacutainer) for peripheral blood mononuclear cell (PBMC) isolation and a 2 mL EDTA tube (BD Vacutainer K2) for hematological parameter analysis. Regarding treatment, the study participants received chloroquine and primaquine according to the dosage recommended by the Ministry of Health guidelines.

A search was conducted in the Epidemiological Surveillance Information System for Malaria (SIVEP-Malaria) to identify previous episodes of malaria and cases of recurrence among participants during the 90 days. SIVEP-Malaria is the official Brazilian system for recording all mandatory notifications of malaria cases. Established by the Health Surveillance Secretariat, SIVEP-Malaria aims to enhance the quality and timeliness of epidemiological data, with a particular focus on the Amazon region. The system enables real-time data entry through an online platform and includes tools for case notification, patient identification, probable infection site registration, and laboratory result tracking.^49^

### *Pv*MSP1_19_ Antigen Production

P. vivax MSP-1 is a polymorphic protein abundantly expressed on the merozoite surface, containing conserved and dimorphic interspecies blocks. During the invasion process, proteolytic cleavage releases most of the molecule from the merozoite surface, and only a 19 kDa fragment of the C-terminal region (*Pv*MSP1_19_) is carried into the invaded erythrocytes.^50^ *Pv*MSP1_19_ antigen was expressed as a glutathione *S*-transferase (GST) fusion protein (*Pv*MSP1_19_-GST), using the gene sequence from the Belém strain. Details of the construct are provided elsewhere.^51^ Briefly, *Pv*MSP1_19_ was amplified by PCR using the original Belém strain clone as a template, encoding amino acids from 1615 to 1726. The recombinant *Pv*MSP1_19_ protein thus encodes 111 amino acids and includes the two EGF-like motifs described in other MSP1 molecules. The amplified fragment was cloned using standard methods and subcloned into the pGEX-3X expression vector. GST was also produced separately as a control. Both recombinant proteins and GST were purified by affinity chromatography on glutathione-Sepharose 4B columns (Pharmacia, Uppsala, Sweden), following established protocols.^51^ Purity was assessed by SDS-PAGE, and protein concentration was measured by using the Bradford assay.

### Laboratory Analyses and Hematological Analysis

A complete blood count was performed for all collected samples to evaluate hematimetric levels, leukocyte counts, and platelet counts using the Sysmex KX-21N analyzer (Sysmex Corporation, Japan).

### Isolation of Peripheral Blood Mononuclear Cells (PBMCs)

The samples collected in heparin tubes were processed for PBMC separation through density gradient centrifugation using Ficoll-Paque Plus (Sigma-Aldrich). The PBMCs were stored at −80 °C, until use, in a cryopreservant solution containing 90% fetal bovine serum (FBS, Gibco, Thermo Scientific) and 10% dimethyl sulfoxide (DMSO). All frozen samples were maintained at over 95% cell viability.

### Cell Culture Assay

Cells were gradually thawed, with 0.5 × 10^6^ cells used per well. Cells were added to a U-bottom 96-well plate (Corning) and cultured in the presence of Roswell Park Memorial Institute medium (RPMI 1640, Gibco19, Thermo Scientific) with 10% FBS at 37 °C, supplemented with brefeldin A (Invitrogen) at a final concentration of 10 μg/mL and Ionomycin (Sigma-Aldrich) at 1 μg/mL. Each well was designated for a specific stimulus: glutathione *S*-transferase (GST) and P. vivax merozoite surface protein-1 (*Pv*MSP1_19_-GST). The viability of cells was assessed under standardized conditions using a negative control without stimulus and a positive control with phorbol 12-myristate 13-acetate (data not shown). PMA was used as a positive control at a final concentration of 25 ng/mL, GST as an antigen control at 10 μg/mL, and *Pv*MSP1_19_-GST as the specific Plasmodium antigen at 20 μg/mL.^52^ The culture conditions were maintained for 24 h in a humidified incubator at 37 °C with 5% CO_2_. An EDTA solution (20 mM) was used to stop cell reactions to the stimuli and prepare for cell immunophenotyping.

The cell cultures were performed in duplicate to ensure reproducibility of the results. First of all, we established cutoff values for baseline IFN-γ production inherent to the manipulation of unstimulated samples (negative control). These values were inserted into the figures: at D0, the cutoff was 618 MFI, while at D90, the median was 493 MFI. The anti-*Pv*MSP1_19_ cellular response was defined based on intracellular IFN-γ production in CD3^+^CD4^+^CD45RO^+^ T cells after *ex vivo* stimulation with the *Pv*MSP1_19_-GST protein and glutathione *S*-transferase (GST), measured by the mean fluorescence intensity (MFI) of the PECy7 fluorochrome conjugated to anti-IFN-γ. Individuals were considered to have an anti-*Pv*MSP1_19_ cellular response when IFN-γ production by CD3^+^CD4^+^CD45RO^+^ cells in response to *Pv*MSP1_19_-GST had a higher MFI than in response to GST. Following data acquisition, the production of intracellular IFN-γ was measured in CD4^+^CD45RO^+^ and CD8^+^CD45RO^+^ T cells cultured in the presence of *Pv*MSP1_19_-GST. To determine whether the intracellular IFN-γ production was specifically directed against *Pv*MSP1_19_, a GST cutoff was calculated using the mean MFI of duplicate CD3^+^CD4^+^CD45RO^+^ and CD8^+^CD45RO^+^ T cells cultured in the presence of GST, plus 2 standard deviations (SD). A cellular response was considered specific to *Pv*MSP1_19_ when the mean MFI of intracellular IFN-γ production in the presence of *Pv*MSP1_19_-GST was greater than the GST cutoff, regardless of whether the absolute IFN-γ levels in response to MSP1_19_ were high or low.

### Immunophenotyping of Leukocyte Subpopulations with Surface and Intracellular Staining

To identify helper T lymphocyte populations and CD4^+^ T cells producing interferon-gamma (IFN-γ) and CD45RO, human monoclonal antibodies (BD Biosciences, San Jose, CA, USA) were used: anti-CD3-FITC, anti-CD4-PERCP, anti-IFN-γ-PECy7, and anti-CD45RO-APC. For cytotoxic CD8^+^ T cells producing IFN-γ and CD45RO, BD human monoclonal antibodies were used: anti-CD8-APC-H7, in addition to anti-CD3, anti-CD45RO, and anti-IFN-γ, as mentioned (Figure 5).

**Figure 5:**
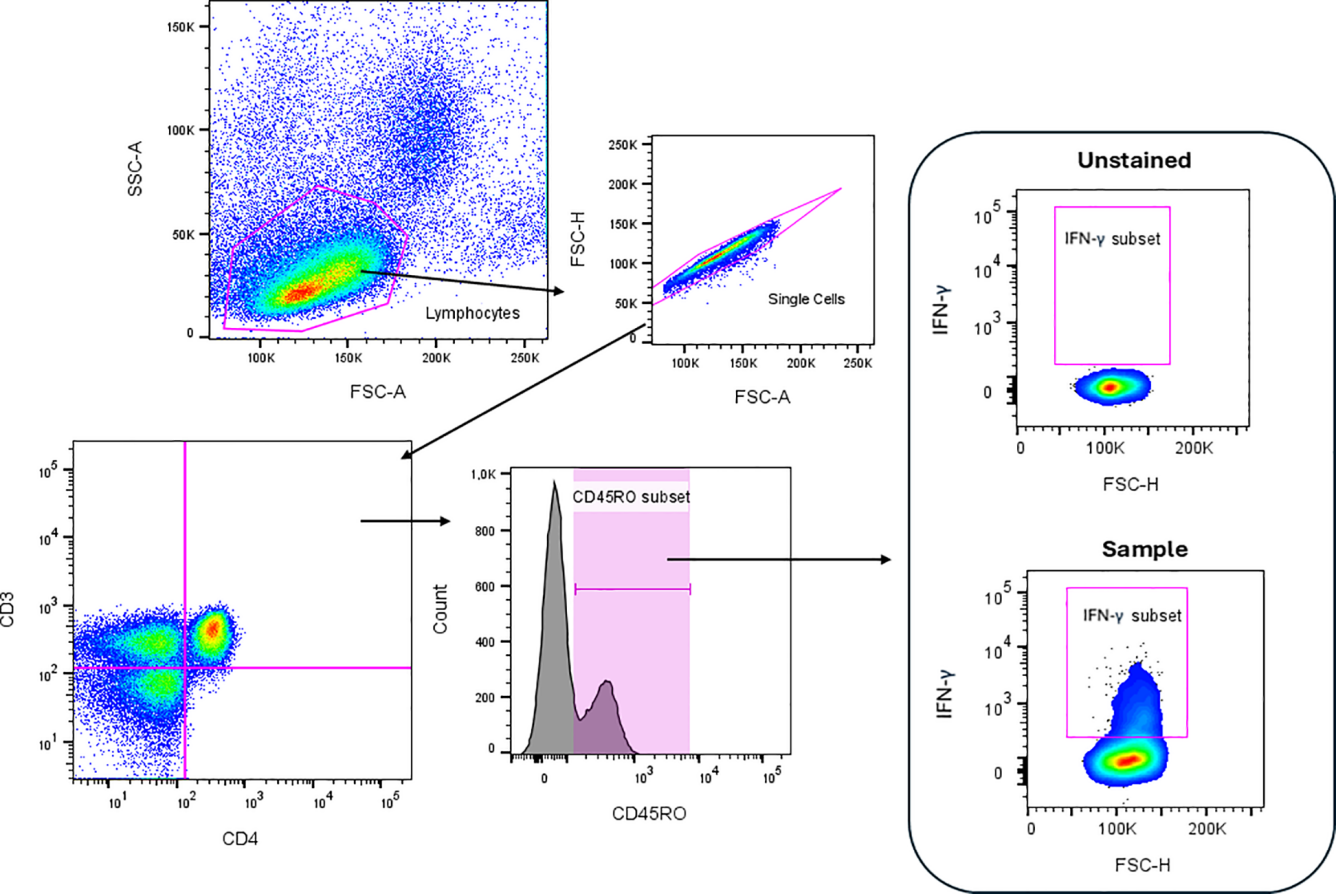
Flow cytometry gating strategy for the detection of CD4^+^CD45RO^+^ and CD8^+^CD45RO^+^ T cells producing IFN-γ. Gating strategy was used to identify CD4^+^ and CD8^+^ T lymphocytes producing IFN-γ and expressing CD45RO. Lymphocytes were initially selected based on their physical properties (FSC vs SSC). From this gate, T cells were defined by CD3 expression. Helper (CD4^+^) and cytotoxic (CD8^+^) T cell subsets were then identified. Within these subsets, memory T cells were selected based on CD45RO expression, and intracellular IFN-γ production was evaluated. The antibodies used were anti-CD3-FITC, anti-CD4-PERCP, anti-CD8-APC-H7, anti-CD45RO-APC, and anti-IFN-γ-PECy7.

After stopping cell stimulation, cells were placed in cytometry tubes for surface staining with monoclonal antibodies and incubated in the dark for 30 min. Erythrocyte lysis was performed by vortex homogenization using 1 mL of lysis solution (0.285 g/L sodium citrate, 5.4 mL/L formaldehyde P.A., 3 mL/L diethylene glycol P.A., and 80 μL/L commercial heparin (5000 UI/mL)). Cells were incubated and washed with phosphate-buffered saline + 0.5% albumin (PBS-W).

For intracellular staining, cells were permeabilized using PBS-P solution (0.5% albumin and 0.5% saponin). Subsequently, samples previously marked with other antibodies were stained intracellularly with anti-IFN-γ. Data acquisition of 100,000 events was performed using the BD FACSCanto II flow cytometer.

### Statistical Analysis

Data from the cytometric immunophenotyping assay were acquired using FlowJo software v. 10.1. Thereafter, the relative fluorescence intensity (RFI) of IFN-γ was determined by dividing the mean fluorescence intensity (MFI) of IFN-γ expressed by *Pv*MSP1_19_-GST-stimulated cells by the MFI of GST-stimulated cells, enabling comparison of fluorescence levels between the two conditions. The Shapiro–Wilk test was used to test the normality of the distribution. Paired *t*-test was used for parametric, and Mann–Whitney test was used for nonparametric comparisons between two groups (D0 and D90). Correlation analyses were conducted by using Pearson correlation methods for continuous data.

Relative fluorescence intensity (RFI) of IFN-γ was determined by dividing the mean fluorescence intensity (MFI) of IFN-γ expressed by *Pv*MSP1_19_-GST-stimulated cells by the MFI of GST-stimulated cells, enabling a comparison of fluorescence levels between the two conditions.

The generalized estimating equations (GEE) model was applied to assess trends in hematological values from D0 to D90. GEE is a statistical modeling approach used to analyze longitudinal or correlated data, particularly when repeated measures are collected from the same subjects over time. In our analysis, data were structured by group (responders and nonresponders), and the model accounted for within-subject correlations to evaluate changes in hemoglobin and platelet levels over time. A summary plot was generated, showing the mean trajectories at both time points, and the model identified significant intergroup differences for these parameters. Unlike traditional regression models that assume independence between observations, GEE accounts for the within-subject correlation structure, allowing for a more robust estimation of population-averaged effects. Data were structured by group (responders and nonresponders), and repeated measures of hematological variables were taken at two time points: D0 (acute phase) and D90 (convalescence). This analysis was performed using Stata v16.0 software. The GEE model was applied to compare these groups over time, evaluating the correlation between repeated observations within individuals. The analysis generated a summary plot depicting mean trajectories for hemoglobin and platelet counts at both time points. The model indicated significant differences between responders and nonresponders, particularly for hemoglobin and platelet levels, highlighting distinct recovery profiles between the groups. Statistical analyses were conducted using GraphPad Prism 9.0 and Stata v16.0 software. Statistical significance was at *p* ≤ 0.05.

## Supplementary Material



## Notes

Comparison of age, days of symptoms, and hematological data between responders and nonresponders on D0 (PDF)

## Notes

Ethical Considerations: Ethical approval for the study was granted by the Ethics Committee of the Dr. Heitor Vieira Dourado Tropical Medicine Foundation (FMT-HVD) under CAEE 39198320.8.0000.0005, in accordance with CNS Resolution No. 466/2012. All participants and their guardians signed the Informed Consent Form (ICF) and, where applicable, the Assent Form.

## Abbreviations

CD3: cluster of differentiation 3
CD4+: cluster of differentiation 4
CD8+: cluster of differentiation 8
CD45RO: cluster of differentiation 45RO
EDTA: ethylenediaminetetraacetic acid
FBS: fetal bovine serum
FMT-HVD: Dr. Heitor Vieira Dourado Tropical Medicine Foundation
G6PD: glucose-6-phosphate dehydrogenase
GEE: generalized estimating equations
GST: glutathione *S*-transferase
IFN-γ: interferon-gamma
IL-1β: interleukin-1 beta
IL-2: interleukin-2
IL-12: interleukin-12
IQR: interquartile range
MCH: mean corpuscular hemoglobin
MCHC: mean corpuscular hemoglobin concentration
MCV: mean corpuscular volume
MFI: mean fluorescence intensity
MPV: mean platelet volume
PBMCs: peripheral blood mononuclear cells
PMA: phorbol 12-myristate 13-acetate
P. falciparum: Plasmodium falciparum
P. vivax: Plasmodium vivax
*Pv*MSP1_19_: Plasmodium vivax merozoite surface protein 1_19_
RBCs: red blood cells
RDW: red cell distribution width
SD: standard deviation
SIVEP-Malaria: Epidemiological Surveillance Information System for Malaria
Th1: T-helper 1
WHO: World Health Organization
