# Supplementary material for: IFN‑γ Production in Memory CD4 + T Cells in Response to MSP119 Antigen and Its Correlation with Anemia and Thrombocytopenia in Pediatric Vivax Malaria
Source: ACS Omega. 2025 Jun 10;10(24):25621–32. doi: 10.1021/acsomega.5c01165 (PMC12199051; doi:10.1021/acsomega.5c01165)
Supplement: Supplementary file 1 [file ao5c01165_si_001.pdf]

IFN-γ production in memory $CD4^+$ T cells in response to $MSP1_{19}$ antigen and its correlation with anemia and thrombocytopenia in pediatric vivax malaria

Ana C. Shuan Laco[1,2], Yury O. Chaves[1,3], Anne C. G. de Almeida[1], Elizangela S. Farias[3], Victor I. Mwangi[2], Marcia V. G. Vallejos[2], Gerhard Wunderlich[5], Paulo A. Nogueira[3]*, Gisely C. de Melo[1,2,4].

[1] Programa de Pós-Graduação em Ciências aplicadas à Hematologia (PPGH-UEA). Universidade do Estado do Amazonas, Manaus, 69050-001, Amazonas, Brazil.

[2] Programa de Pós-Graduação em Medicina Tropical (PPGMT-UEA); Universidade do Estado do Amazonas, Manaus, 69040-000, Amazonas, Brazil.

[3] Fundação Oswaldo Cruz (FIOCRUZ); Instituto Leônidas & Maria Deane, (ILMD/Fiocruz-Amazônia); Manaus, 69057-070, Amazonas, Brazil

[4] Fundação de Medicina Tropical Doutor Heitor Vieira Dourado. Manaus, 69050-001, Amazonas, Brazil.

[5]Instituto de Ciências Biomédicas da Universidade de São Paulo, São Paulo, 05508-000, Brazil.

*Corresponding author

SUPPLEMENTARY FIGURE

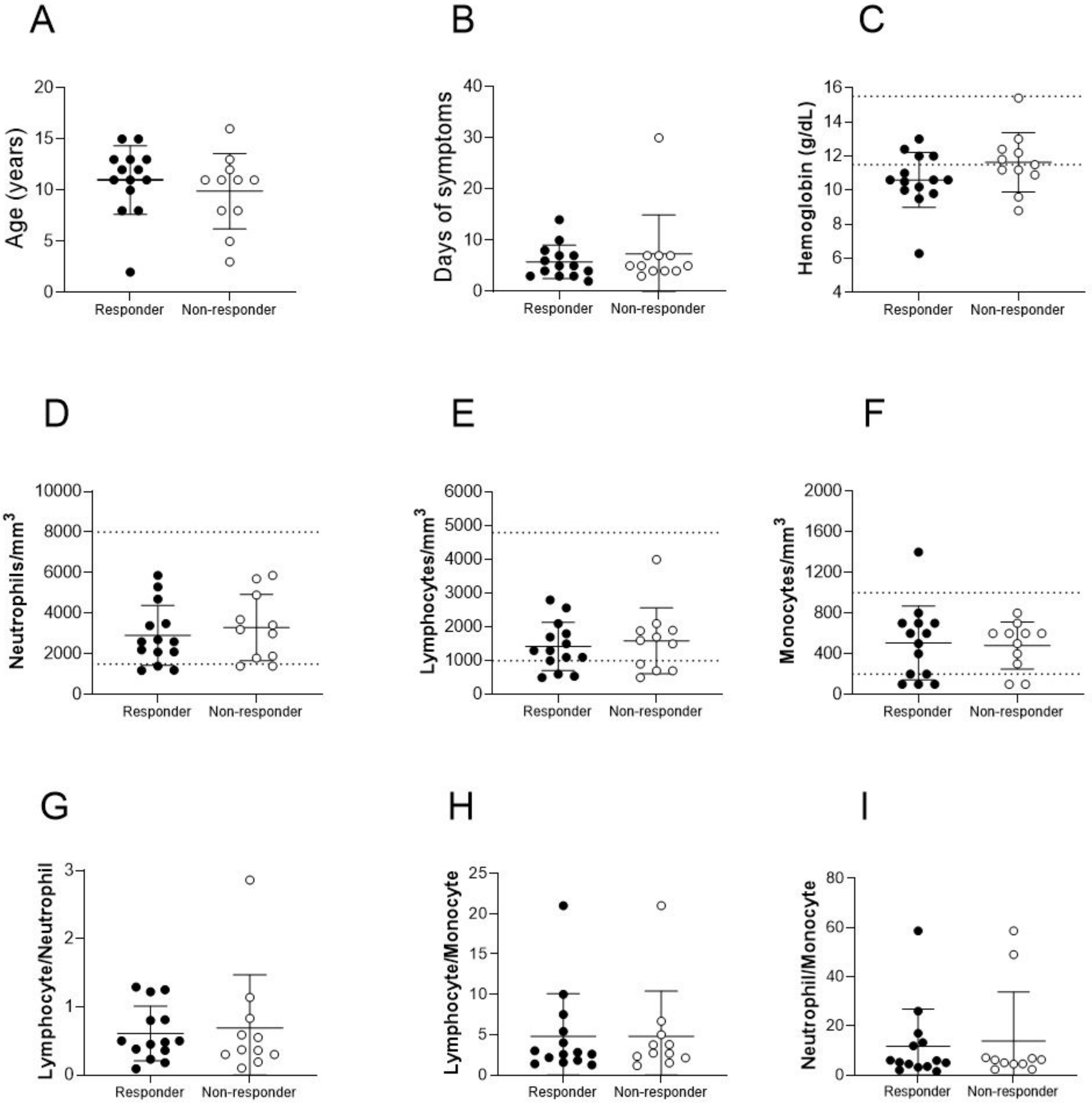

Supplementary Figure 1. Comparison of age, days of symptoms and hematimetric data between Responders and Non-Responders on D0. Each gray dot represents an individual patient's age, days of symptoms and hematimetric data of responders and non-responders, with box plots showing the median, interquartile range, and overall value distribution within each group. No difference is found based on a statistical comparison using the Mann-Whitney test. A) age; B) days of symptoms; C) hemoglobin; D) Neutrophils; E) Lymphocytes; F) Monocytes; G) Lymphocytes/Neutrophils ratio; H) Lymphocytes/Monocytes ratio; I) Neutrophils/Monocytes ratio.
